# Supplementary material for: Characterizing Structural and Kinetic Ensembles of Intrinsically Disordered Proteins Using Writhe
Source: J Chem Theory Comput. 2025 Nov 19;21(23):12289–303. doi: 10.1021/acs.jctc.5c01133 (PMC12874386; doi:10.1021/acs.jctc.5c01133)
Supplement: Supplementary file 1 [file ct5c01133_si_001.pdf]

Supplementary Information

Characterizing structural and kinetic ensembles of intrinsically disordered proteins using writhe

Thomas R. Sisk[1], Simon Olsson[2], Paul Robustelli[1†]

[1] Dartmouth College, Department of Chemistry, Hanover, NH, 03755
[2] Department of Computer Science and Engineering, Chalmers University of Technology and University of Gothenburg, SE-41296 Gothenburg, Sweden

† To whom correspondence should be addressed.

Paul Robustelli:

E-mail: Paul.J.Robustelli@Dartmouth.edu

Gaussian integrals and writhe of continuous curves.

The writhe of continuous curves in $\mathbb{R}^3$ can be expressed as the Gaussian integral[1, 2],

$$\frac{1}{4\pi}\int_0^{L_1}\int_0^{L_2}\frac{\boldsymbol{T}(s_1)\times\boldsymbol{T}(s_2)\cdot\left(\boldsymbol{r}(s_1)-\boldsymbol{r}(s_2)\right)}{\|\boldsymbol{r}(s_1)-\boldsymbol{r}(s_2)\|^3}\,ds_1\,ds_2, \tag{1}$$

where $\boldsymbol{r}(s)$ is a vector valued function giving the position of each point along a space curve as a function of the arc length parameter s, which takes values in the interval [0, L] and spans length of the curve, L. The function $\boldsymbol{T}(s)$ is the unit tangent vector at s, $\boldsymbol{T}(s) = d\boldsymbol{r}/ds$, which describes the local direction of the curve. The parameters s_1 and s_2 are points on the space carve, and serve as integration variables, such that the double integral in (1) runs over all pairs of points. Evaluating the expression under the integral for two arbitrary points on the curve as a function of their arclengths (s_1, s_2) and inserting the definitions of the cross and dot products yields:

(2.a) $\boldsymbol{r}(s_1)-\boldsymbol{r}(s_2)=\Delta\boldsymbol{r}$

(2.b) $\boldsymbol{T}(s_1)\times\boldsymbol{T}(s_2) = \hat{\boldsymbol{n}}\sin\theta$ $\quad\Longrightarrow\quad$ $\dfrac{\boldsymbol{T}(s_1)\times\boldsymbol{T}(s_2)\cdot\Delta\boldsymbol{r}}{\|\Delta\boldsymbol{r}\|^3}=\dfrac{\sin\theta\hat{\boldsymbol{n}}\cdot\Delta\boldsymbol{r}}{\|\Delta\boldsymbol{r}\|^3}=\dfrac{\sin\theta\cos\phi}{\|\Delta\boldsymbol{r}\|^2}.$ (2)

(2.c) $\hat{\boldsymbol{n}}\cdot\Delta\boldsymbol{r} = \|\Delta\boldsymbol{r}\|\cos\phi$

In the above, we have abbreviated the displacement vector between the two points on the curve as $\Delta\boldsymbol{r}$ (2.a). We use the typical definition of the cross product of unit vectors (2.b) to write $\boldsymbol{T}(s_1)\times\boldsymbol{T}(s_2)$ as a unit vector ($\hat{\boldsymbol{n}}$) orthogonal to the plane containing $\boldsymbol{T}(s_1)$ and $\boldsymbol{T}(s_2)$ multiplied by the sine of the angle between $\boldsymbol{T}(s_1)$ and $\boldsymbol{T}(s_2)$, which we denote as θ. Additionally, we use the typical definition of the dot product (2.c) to write $\hat{\boldsymbol{n}}\cdot\Delta\boldsymbol{r}$ as the magnitude of $\Delta\boldsymbol{r}$ (distance between points on the curve) multiplied by the cosine of the angle between $\hat{\boldsymbol{n}}$ and $\Delta\boldsymbol{r}$ which we denote as ϕ.

The final expression on the right-hand side of (1) illustrates that the magnitude of the angular component of the writhe ($\sin\theta\cos\phi$) reaches its maximum when the tangent vectors are orthogonal ($\theta = \pm\frac{\pi}{2}$) and their displacement ($\Delta\boldsymbol{r}$) aligns with the normal ($\hat{\boldsymbol{n}}$) of the tangent plane ($\phi = 0, \pi$). This occurs when $\boldsymbol{T}(s_1)$, $\boldsymbol{T}(s_2)$ and $\Delta\boldsymbol{r}$ are mutually orthogonal. We also see that the radial component ($1/\|\Delta\boldsymbol{r}\|^2$) causes the writhe to decay quadratically with the Euclidean distance between crossings. The full Gauss integral can be interpreted as the average number of signed crossings taken over all possible viewing angles in 3-space.[1, 3] The integral in equation 1 of the main text can also be written as a function of two curves to define their *Gaussian linking number*.[2, 4]

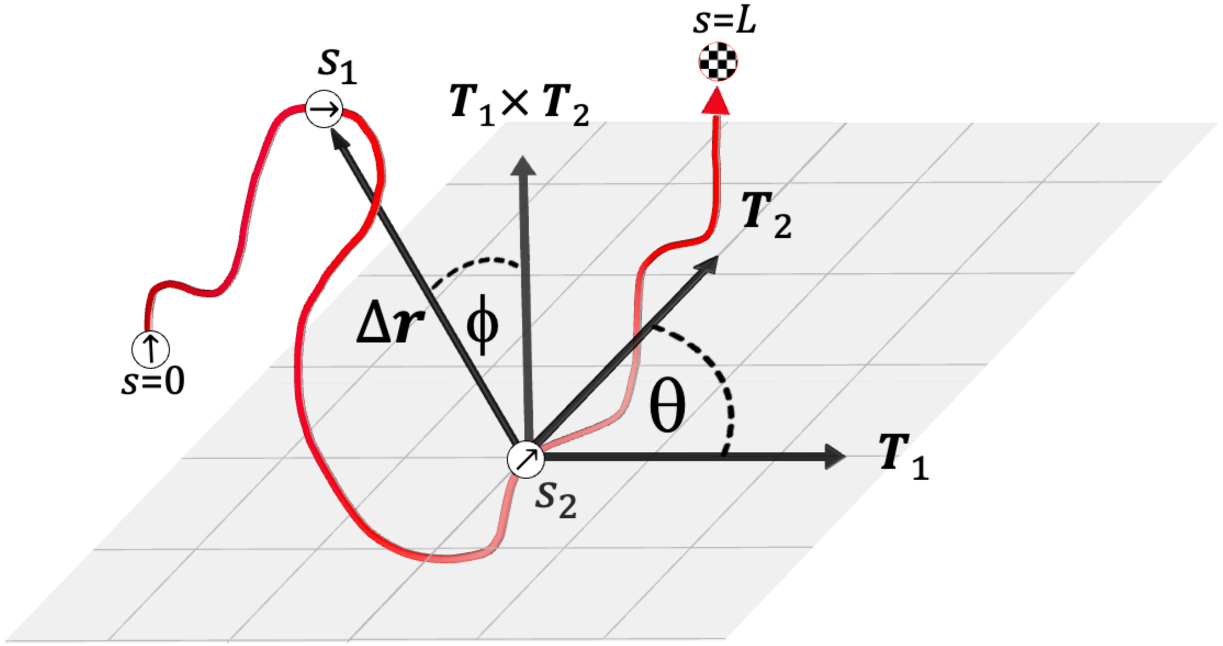

Figure S1: The writhe of a pair of points on a continuous space curve. We illustrate the vectors and angles that characterize the writhe for two points (s_1 and s_2) on a continuous curve parameterized by the arclength, s. Here, we abbreviate the tangents at s_1 and s_2 as $\boldsymbol{T}_1$ and $\boldsymbol{T}_2$ and label the normal vector to the plane containing both tangents as their cross product, $\boldsymbol{T}_1 \times \boldsymbol{T}_2$. The direction of the tangents to the curve at $s = 0$, s_1, and s_2 are also shown as small black arrows inside white circles denoting their position on the curve. The end of the curve is labeled as $s = L$, where L denotes the length of the curve. In this diagram, angle θ between the tangent vectors, $\boldsymbol{T}_1$ and $\boldsymbol{T}_2$, is $\frac{\pi}{2}$ radians or 90°, which maximizes its contribution to the angular component of the writhe. For the above diagram, the writhe monotonically increases as $\phi \to 0°$and $||\Delta \boldsymbol{r}|| \to 0$, i.e., as the points on the curve come close and $\Delta \boldsymbol{r}$ aligns with $\boldsymbol{T}_1 \times \boldsymbol{T}_2$, the tangent vectors appear they're crossing over a larger range of viewing angles or perspectives.

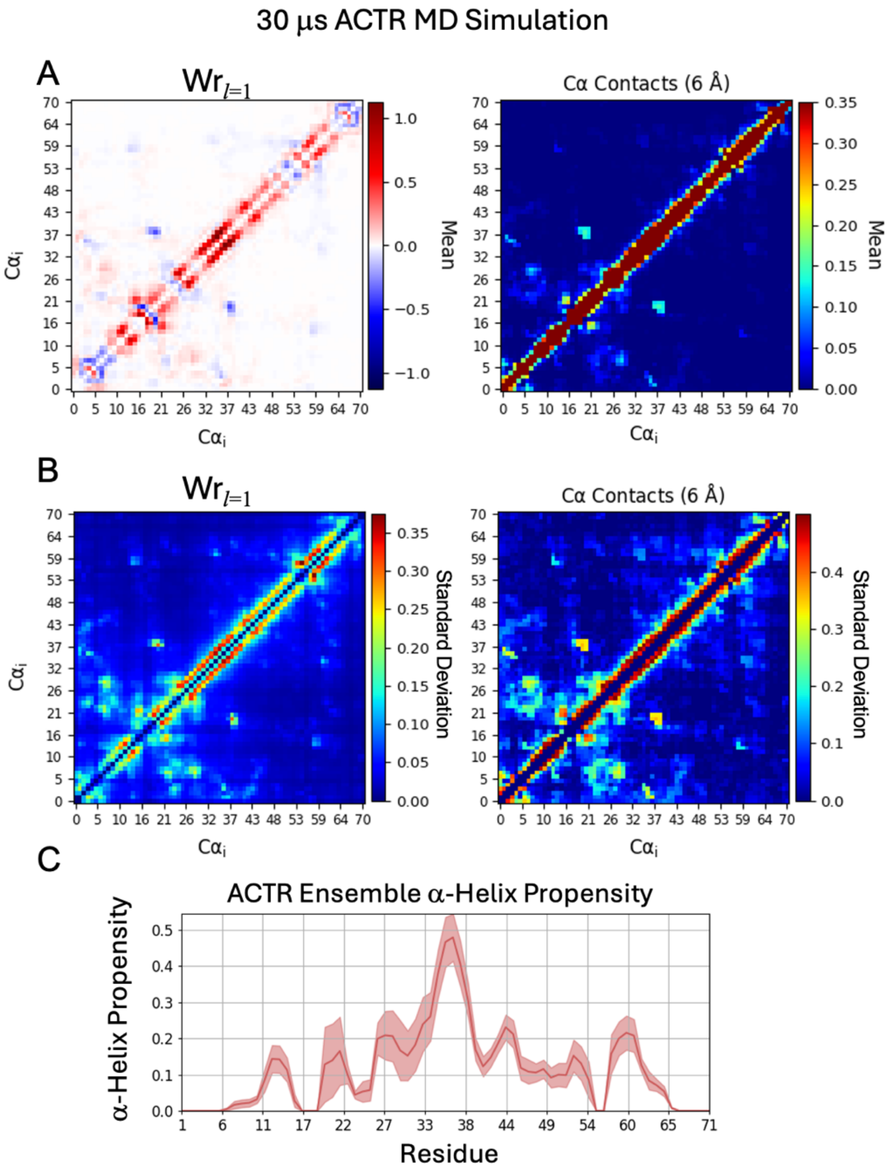

Figure S2: Comparison of the ensemble averages and of pairwise writhe features and Cα contacts observed in a 30μs MD simulation of ACTR. Averages **(A)** and fluctuations **(B)** of the writhe (normalized) and intramolecular contacts between all pairs of Cα atoms in a 30μs MD simulation of the 71-residue IDP ACTR.[5] Intramolecular contacts were defined as occurring in all frames where the minimum distance between the Cα atoms of two residues was less than 6.0 Å. **(C)** Population of a α-helical elements computed using the DSSP algorithm.

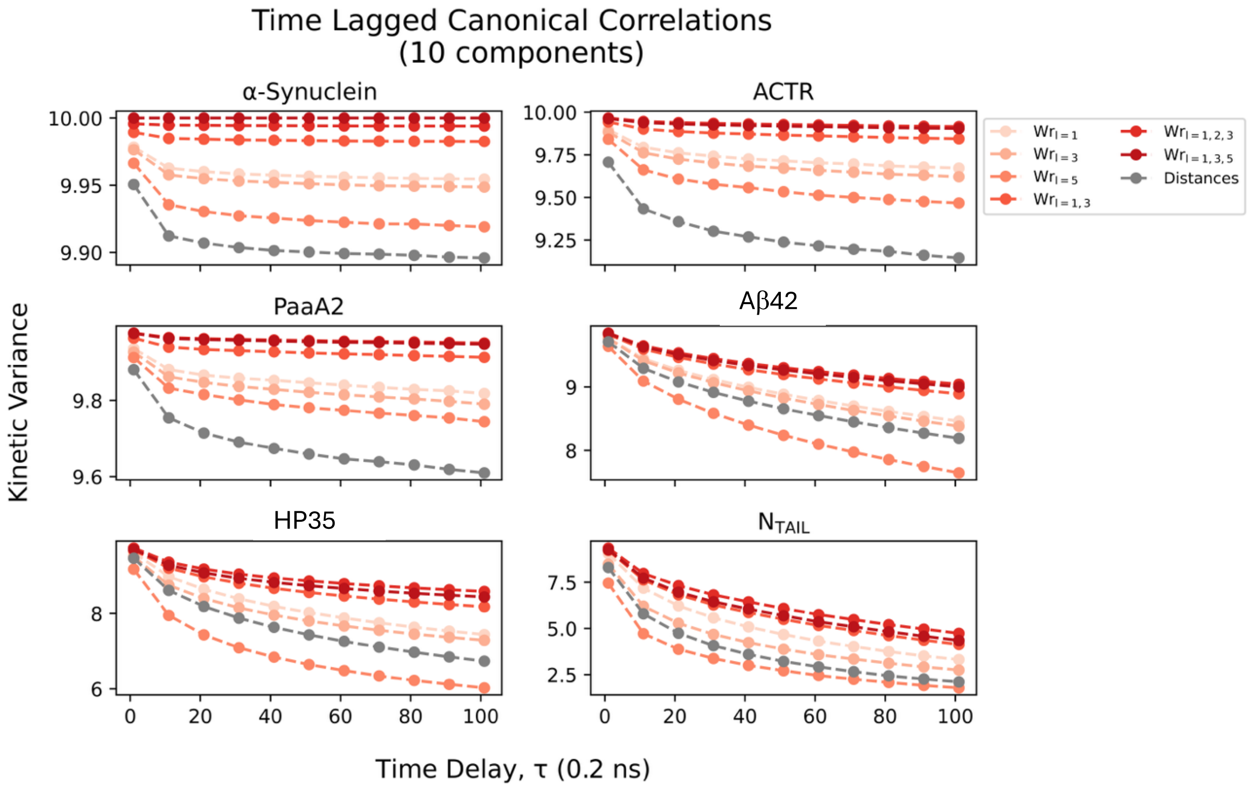

Figure S3: Comparison of the kinetic variances (VAMP-2 scores) of writhe and Euclidean distance features from 6 long-timescale molecular dynamics simulations. Kinetic variances obtained from performing time-lagged canonical correlation analysis[6, 7] (tCCA) for a collection of writhe feature sets (reds) and inter-residue Euclidean distances (grey) from long-timescale molecular dynamics simulations of full-length α-synuclein (140 residues), ACTR (71 residues), PaaA2 (71 residues), Aβ42 (42 residues), HP35 (35 residues) and N_{TAIL} (21 residues). Here, we compute the kinetic variance from the first 10 tCCA components, making the highest possible score 10. The VAMP-2 is truncated to 10 dimensions to negate differences in the dimensionalities of the features sets being compared (each tCCA mode is linearly independent).

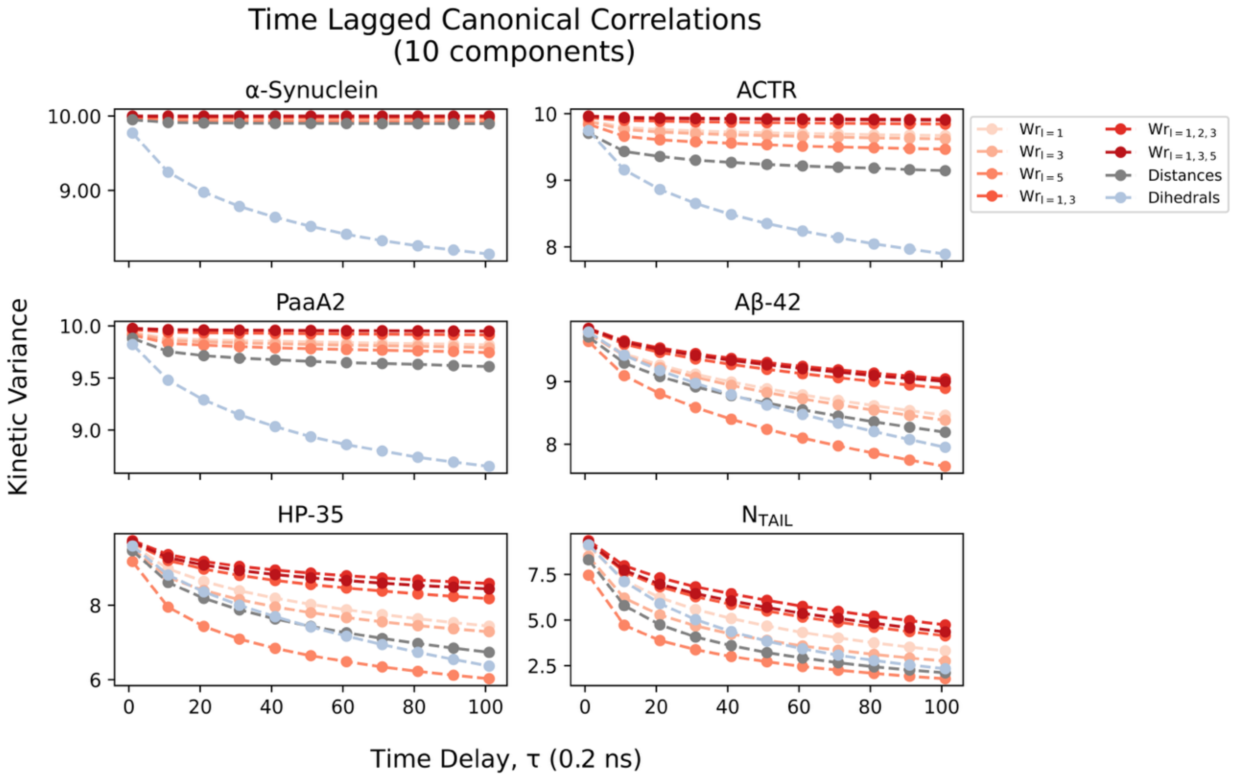

Figure S4: Comparison of the kinetic variances (VAMP-2 scores) of writhe, Euclidean distance and dihedral angle features from 6 long-timescale molecular dynamics simulations. Here, we show the results of Figure S3 with the addition of VAMP-2 scores obtained using dihedral angles. To prevent artifacts resulting from the periodicity of dihedral angles, we performed tCCA using both the sine and cosine of the backbone dihedral angles ϕ and ψ as inputs for tCCA. For each system examined, we observe that tCCA performed using $Wr_{l=1}$ captures more kinetic variance than distance and dihedral features. We observe that dihedral angles, which are inherently local descriptors, yield increasingly lower VAMP-2 scores relative to writhe and distance features as the length of IDPs increase.

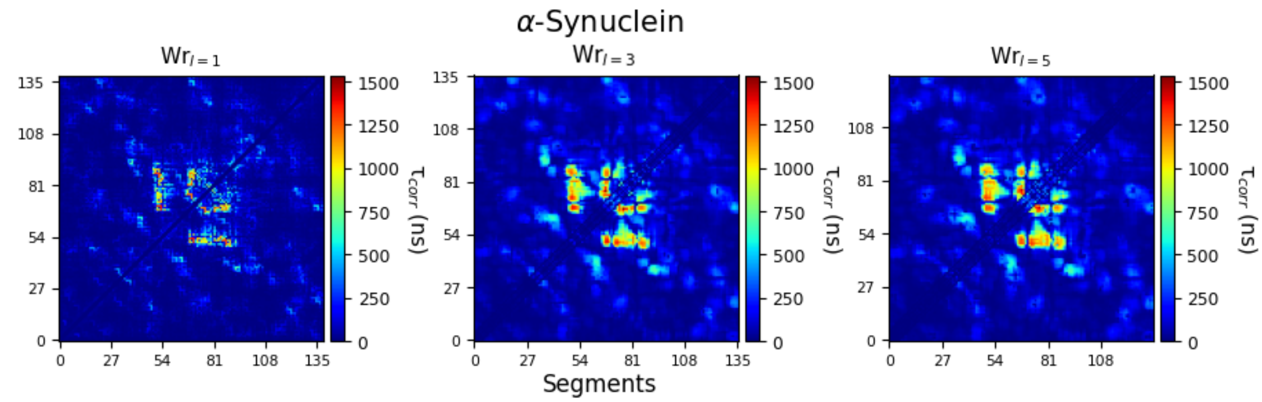

Figure S5. Autocorrelation time (τ_{corr}) matrices for pairwise writhe values between segments, computed at segment lengths of 1, 3 and 5 for a continuous long-timescale molecular dynamics simulation of α-synuclein. The autocorrelation times presented here are estimated from integrating the individual autocorrelation functions of each writhe feature up to the time that they first cross zero.

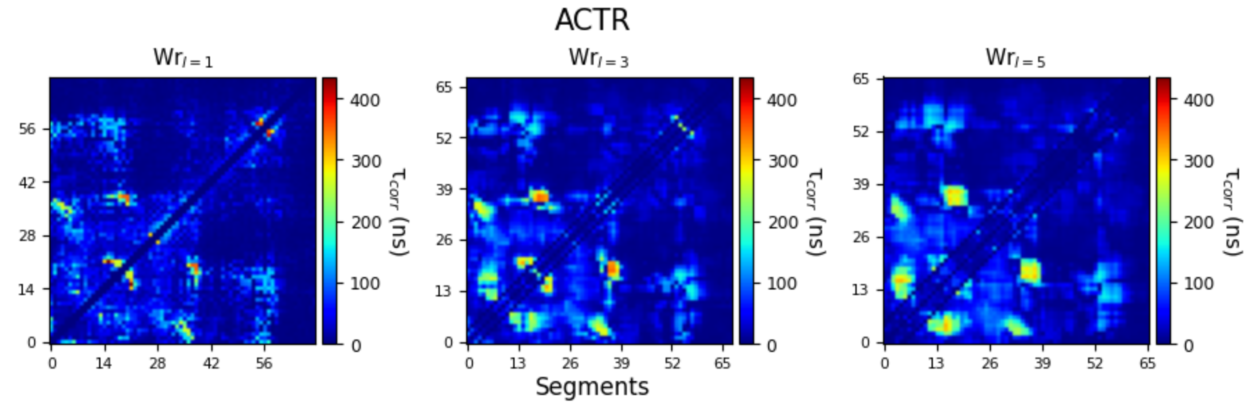

Figure S6. Autocorrelation time (τ_{corr}) matrices for pairwise writhe values between segments, computed at segment lengths of 1, 3 and 5 for a continuous long-timescale molecular dynamics simulation of ACTR. The autocorrelation times presented here are estimated from integrating the individual autocorrelation functions of each writhe feature up to the time that they first cross zero.

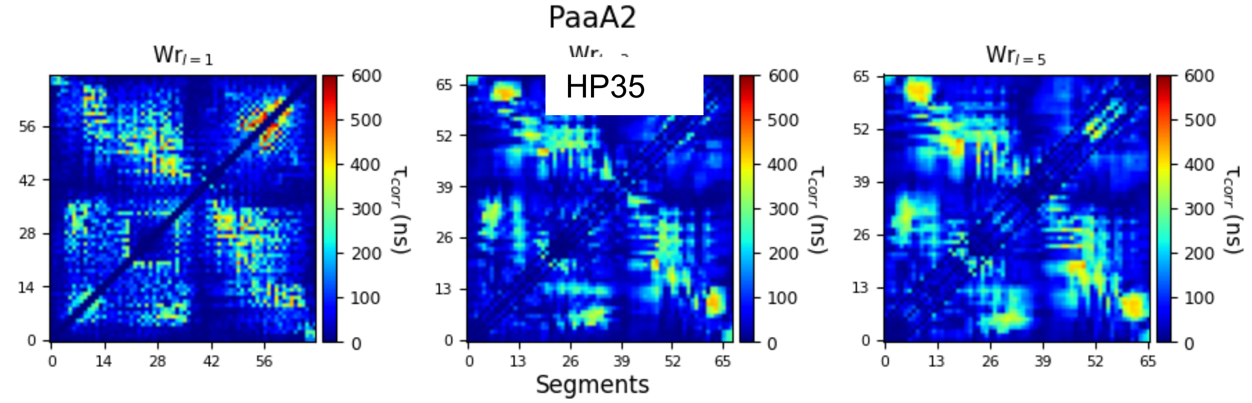

Figure S7. Autocorrelation time (τ_{corr}) matrices for pairwise writhe values between segments, computed at segment lengths of 1, 3 and 5 for a continuous long-timescale molecular dynamics simulation of PaaA2. The autocorrelation times presented here are estimated from integrating the individual autocorrelation functions of each writhe feature up to the time that they first cross zero.

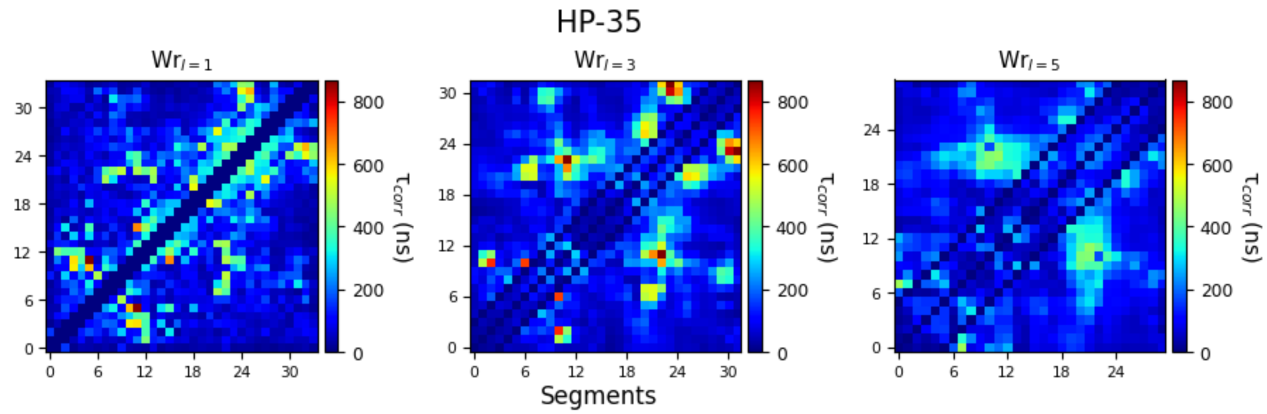

Figure S8. Autocorrelation time (τ_{corr}) matrices for pairwise writhe values between segments, computed at segment lengths of 1, 3 and 5 for a continuous long-timescale molecular dynamics simulation of HP35. The autocorrelation times presented here are estimated from integrating the individual autocorrelation functions of each writhe feature up to the time that they first cross zero.

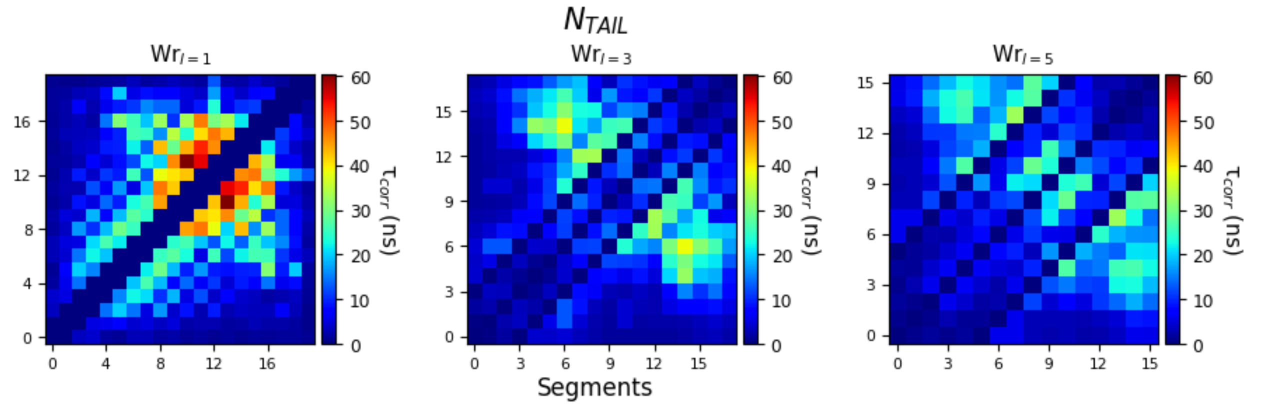

Figure S9. Autocorrelation time (τ_{corr}) matrices for pairwise writhe values between segments, computed at segment lengths of 1, 3 and 5 for a continuous long-timescale molecular dynamics simulation of N_{TAIL}. The autocorrelation times presented here are estimated from integrating the individual autocorrelation functions of each writhe feature up to the time that they first cross zero.

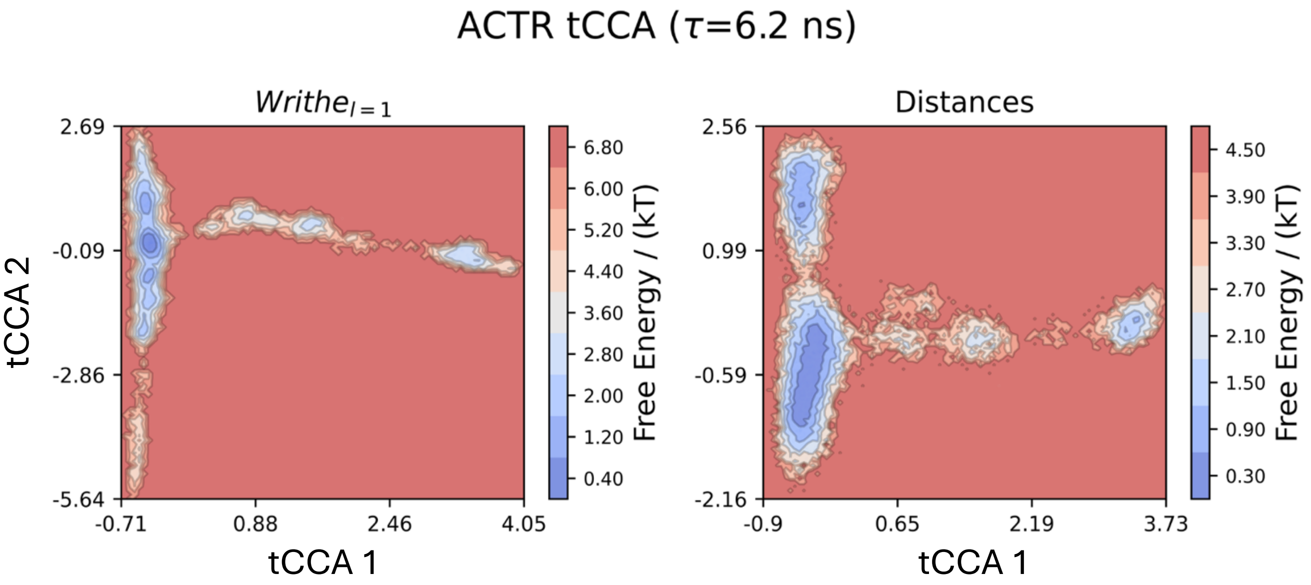

Figure S10. Time-lagged canonical correlation analysis of a long-timescale molecular dynamics simulation of the intrinsically disorder protein, ACTR . We compare free energy surfaces obtained from performing time-lagged canonical correlation analysis (tCCA) on writhe features computed at segment length 1 (**left**) and inter-residue Euclidean distances (**right**) from a continuous, unbiased 30 μs molecular dynamics simulation of ACTR.

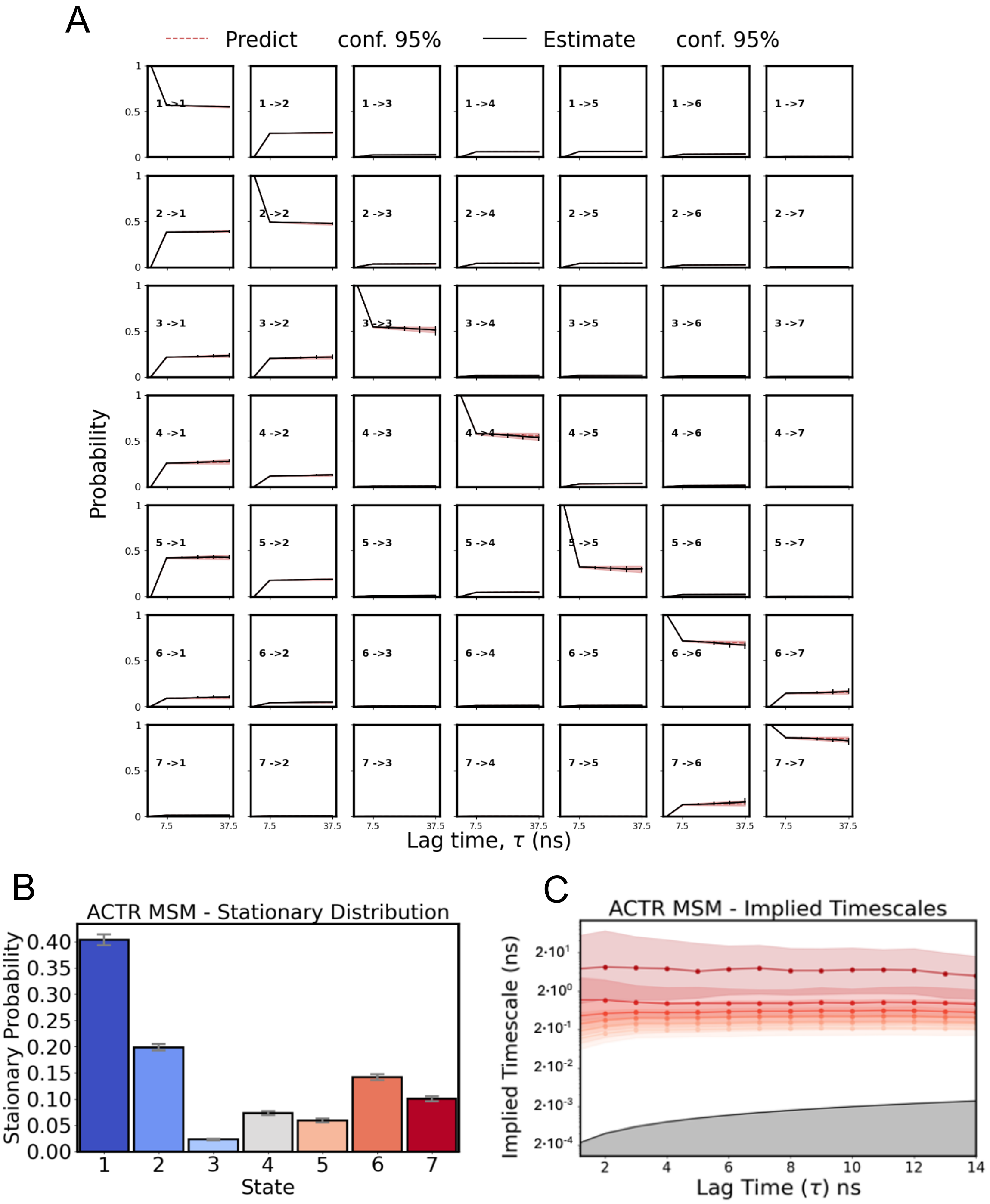

Figure S11. **ACTR writhe MSM validation tests and stationary distribution. (A)** The Chapman-Kolmogrov (CK) test calculated for the MSM of ACTR constructed from multiscale writhe features computed using segment lengths 1, 3 and 5 ($Wr_{l=1,\ 3,\ 5}$). Writhe features were projected onto a 3-dimensional tCCA space and clustered into 40 microstates using the Kmeans algorithm. The CK test evaluates the dependence of an MSMs predictions on the chosen lag time by comparing the evolution of transition probabilities for each state, i, to every other state, j, for integer multiples of the initial lag time of the model (6 ns). Here, we perform the CK test in the space of 7 macrostates obtained from PCCA++ spectral clustering. Red dotted lines ("Predict")

represent transition probabilities predicted by propagating the transition matrix and the solid black lines ("Estimate") indicate transition probabilities obtained from transition matrices resampled from the trajectory data at integer multiples of the lag time. The red shaded region indicates the 95% confidence interval of the mean obtained from Gibbs sampling with 1000 samples. **(B)** The stationary distribution for each state of the MSM with error bars showing the deviation of the 95% confidence interval of the mean. **(C)** The log scaled implied timescales obtained from the MSM transition matrices estimated at increasing lag times. The colored shaded regions show the deviation of the 95% confidence interval of the mean of the implied timescales estimated for each lag time. The solid black line and gray shaded region indicate time scales equal to or less than the lag time and represents the threshold for the fastest ITS that can be resolved by the model.

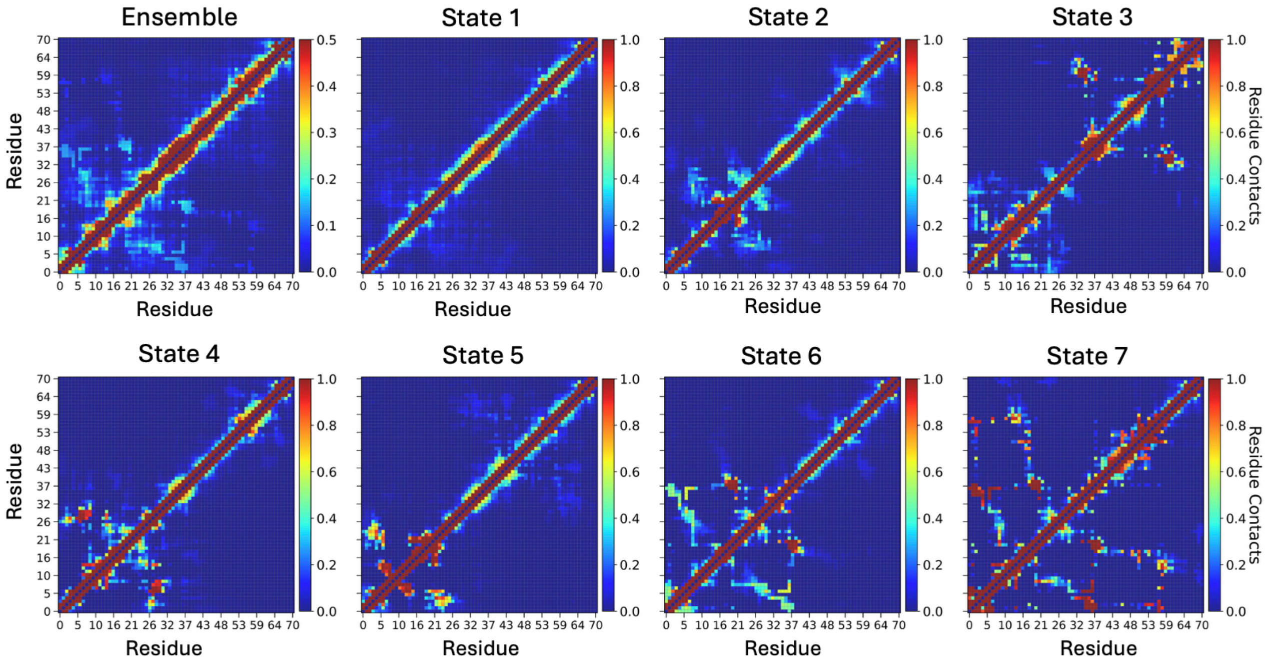

Figure S12. **Intramolecular contact populations in each metastable state of the ACTR writhe MSM.** Intermolecular contacts were defined as occurring in all frames where the minimum distance between the Cα atoms of two residues was less than 6.0 Å. Here, averages are weighted by the predicted equilibrium distribution obtained from the MSM.

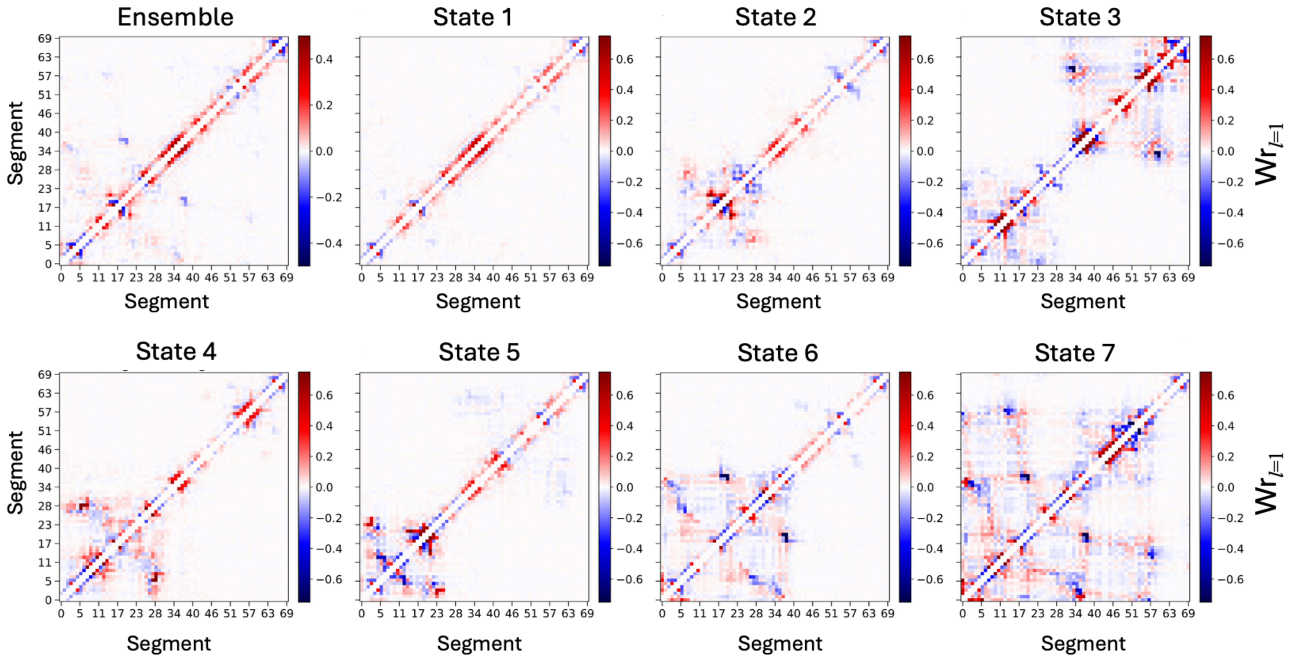

Figure S13. **State-averaged writhe matrices of each metastable state of the ACTR writhe MSM.** The writhe is computed using segment length 1 (Wr$_{l=1}$) and scaled to the interval [-1, 1]. Here**,** averages are weighted by the predicted equilibrium distribution obtained from the MSM.

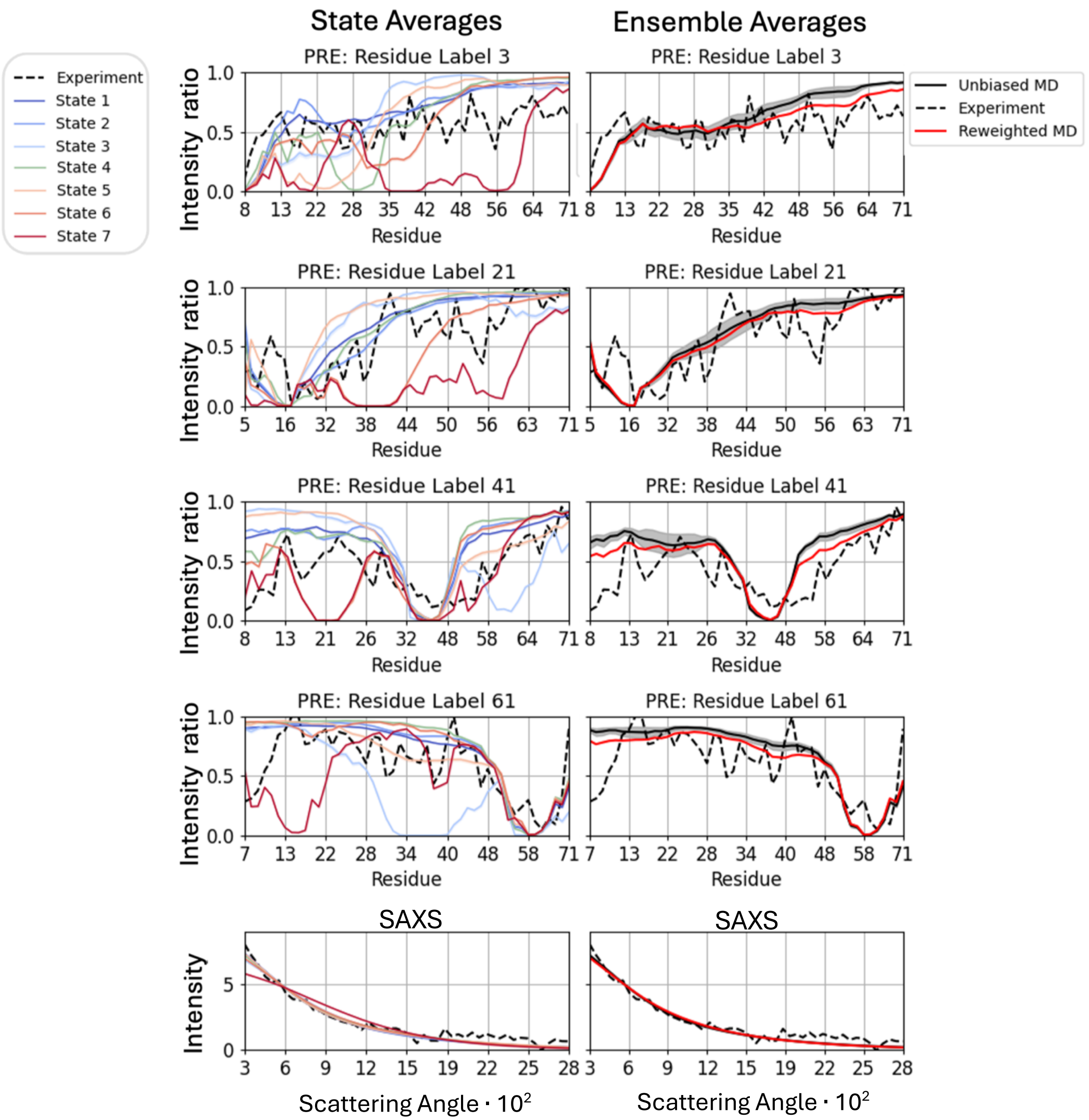

Figure S14: Comparison of predicted and experimental observables obtained from a 30 μs MD simulation of ACTR. We compare experimental observables calculated from each macrostate in the ACTR writhe MSM (left column). We compare the experimental observables calculated from the full unbiased MD trajectory (solid black line), experimental observables calculated from a maximum entropy reweighted trajectory obtained using NMR chemical shifts, PREs, residual dipolar couplings (RDCs) and SAXS data as restraints[8] and experimental values (dotted black line) (right column).

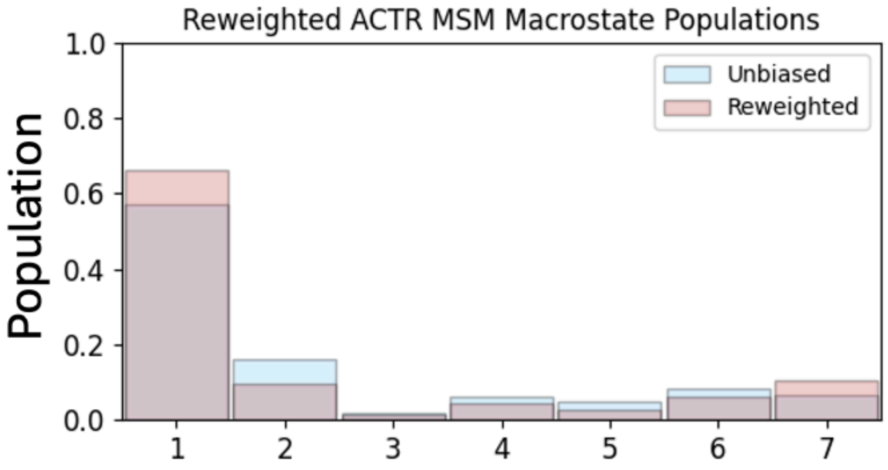

Figure S15: Comparing macrostate populations of the ACTR writhe MSM before and maximum entropy reweighting. We compare the populations of each macrostate of the ACTR writhe MSM before and after performing maximum entropy reweighting with NMR chemical shifts, PREs, residual dipolar couplings (RDCs) and SAXS data as restraints.[8] Populations were determined by the statistical weight of each frame assigned to each macrostate before and after reweighting.

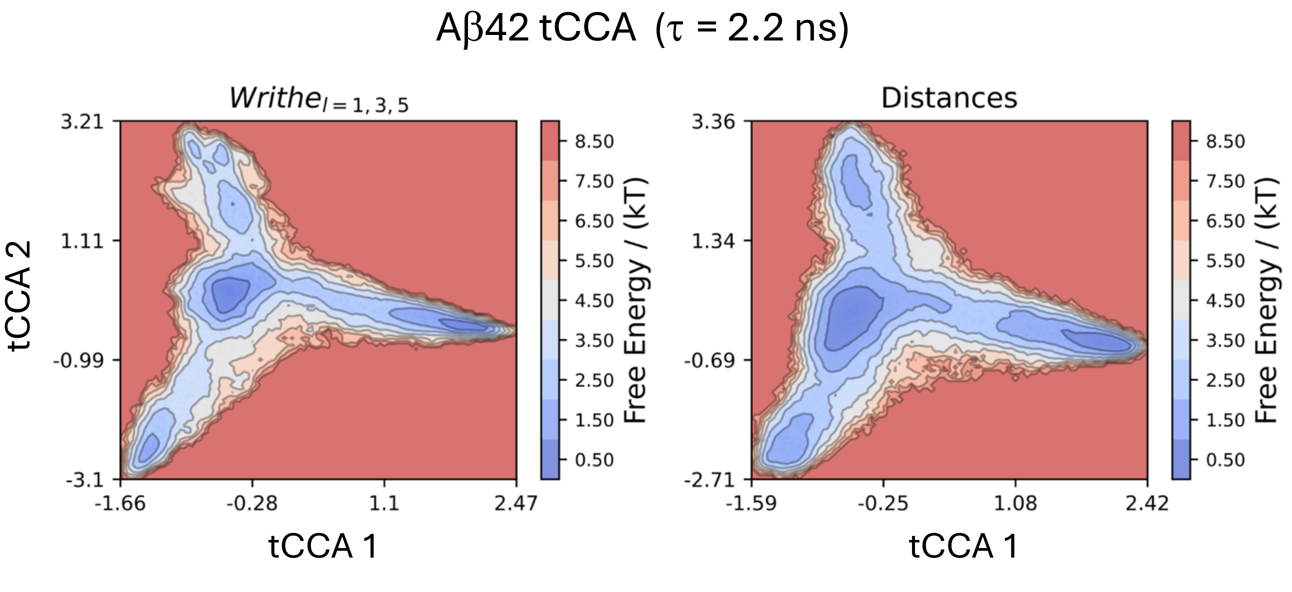

Figure S16. Time-lagged canonical correlation analysis of a long-timescale molecular dynamics simulation of the intrinsically disorder protein, Aβ42. We compare 2D free energy surfaces obtained from performing time-lagged canonical correlation analysis (tCCA) on multiscale writhe features computed using segment lengths 1, 3 and 5 (**right**) and inter-residue distances (**left**) from 315 μs of molecular dynamics simulation of Aβ42.

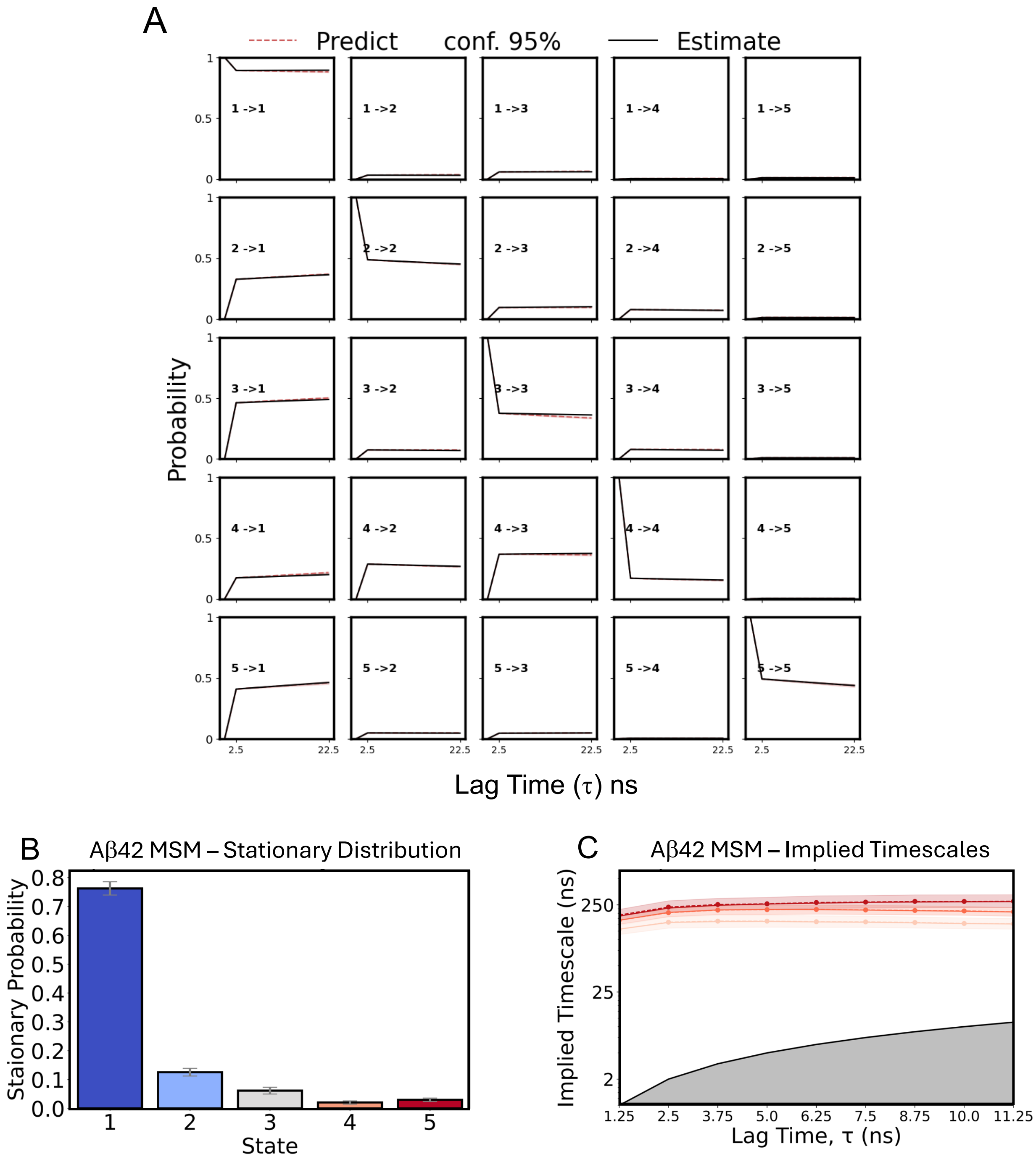

Figure S17. **Aβ42 MSM validation tests and stationary distribution built from multiscale writhe features. (A)** The Chapman-Kolmogrov (CK) test for the MSM of Aβ-42 constructed from multiscale writhe features computed using segment lengths 1, 3 and 5 ($Wr_{1, 3, 5}$). Writhe features were projected into a 3-dimensional tCCA space and clustered into 40 microstates using the Kmeans algorithm. The CK test evaluates the dependence of an MSMs predictions on the chosen lag time by comparing the evolution of transition probabilities for each state, i, to every other state, j, for integer multiples of the initial lag time of the model (2.5 ns). Here, we perform the CK test

in the space of 5 macro-states obtained from PCCA++ spectral clustering. Red dotted lines ("Predict") represent transition probabilities predicted by propagating the transition matrix and the solid black lines ("Estimate") indicate transition probabilities obtained from transition matrices resampled from the trajectory data at integer multiples of the lag time. The red shaded region indicates the 95% confidence interval of the mean obtained from Gibbs sampling with 1000 samples. **(B)** The stationary distribution for each state of the MSM with error bars showing the deviation of the 95% confidence interval of the mean. **(C)** The log scaled implied timescales obtained from the MSM transition matrices estimated at increasing lag times. The colored shaded regions show the deviation of the 95% confidence interval of the mean of the implied timescales estimated for each lag time. The solid black line and gray shaded region indicate time scales equal to or less than the lag time and represents the threshold for the fastest ITS that can be resolved by the model.

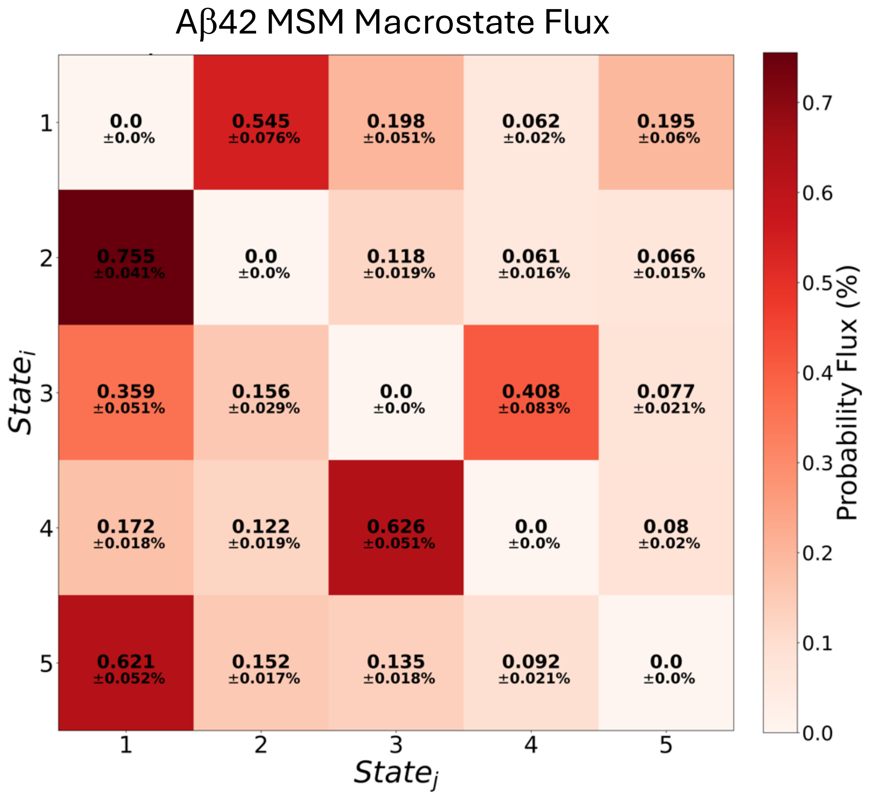

Figure S18. Aβ42 MSM macrostate probability flux matrix for lag time τ = 2.5 ns. The matrix of pairwise total probability fluxes between metastable sets determined from PCCA++ spectral clustering of a 40 microstate MSM of Aβ42 constructed from multiscale writhe features computed from segment lengths 1, 3 and 5 ($Wr_{1, 3, 5}$) and estimated at lag time, τ = 2.5 ns. The probability flux describes the flow of probability mass and can be interpreted as the number of transition events along a certain pathway per time unit. Here, fluxes are normalized by the total outgoing flux from each state. The flux matrix shown here is the bootstrap mean of results obtained from Gibbs sampling of the corresponding MSM transition matrix using 1000 samples. Errors report the mean of the upper and lower deviations of the 95% confidence interval of the bootstrap mean.

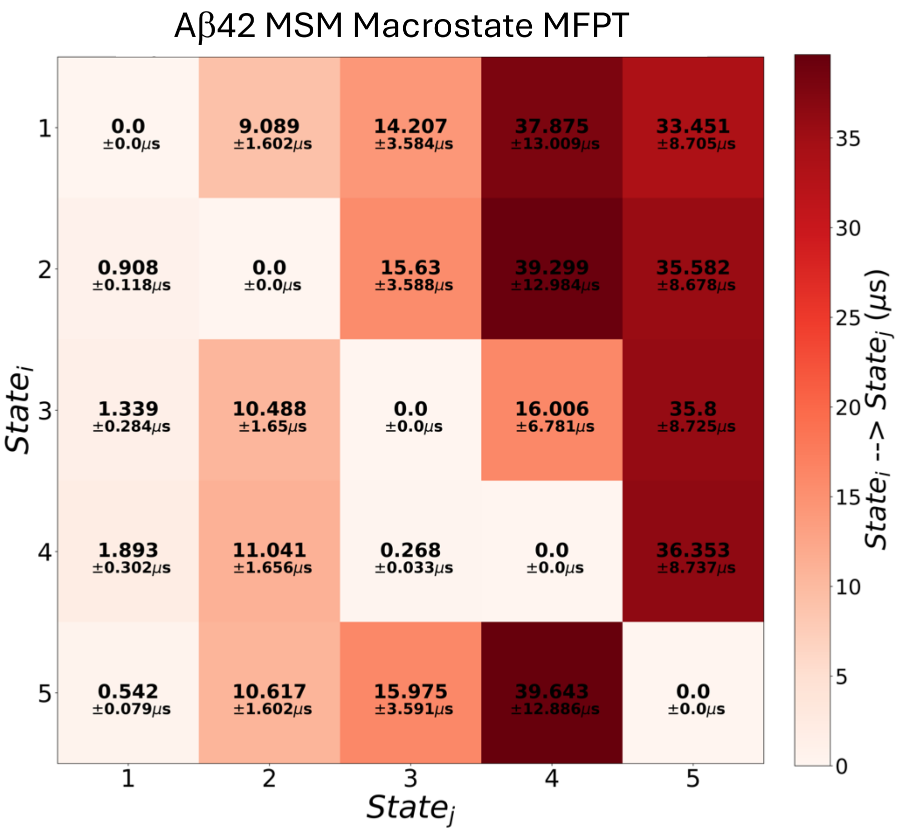

Figure S19. Aβ42 MSM mean first passage time matrix. The mean first passage time (MFPT) matrix shows the average time duration it takes to transition between metastable sets of microstates in the MSM of Aβ42 constructed from multiscale writhe features computed from segment lengths 1, 3 and 5 ($Wr_{l=1,3,5}$). We compute the mean first passage times using the transition matrix and stationary probabilities of the Aβ42 MSM estimated at lag time, $\tau = 2.5$ ns. Errors report the mean of the upper and lower deviations of the 95% confidence interval calculated from Gibbs sampling of the transition matrix using 1000 samples.

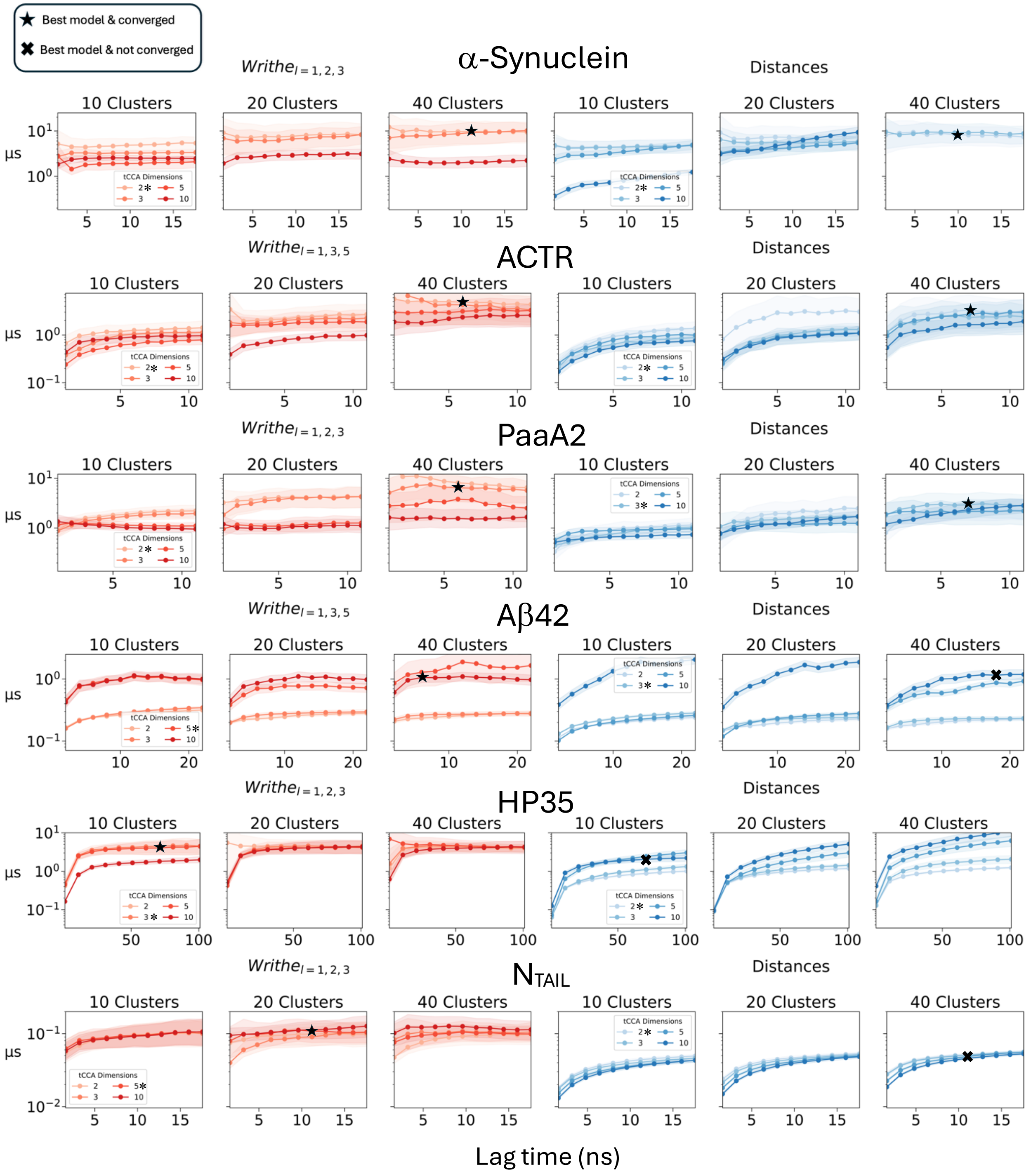

Figure S20. Longest Implied timescales of MSMs as a function of the tCCA dimension and number of clusters for writhe features and inter-residue Euclidean distances. The longest implied timescale (ITS) of an MSM indicates the relaxation time of the slowest dynamical process it captures. Plotting the ITS as a function of the lag time is a validation measure for MSMs – predicted timescales should be approximately invariant over a range of lag times as they correspond to physical observables. Thus, if the predicted ITS for a set hyperparameters fails to produce consistent ITS over a range of lag times, the set of hyperparameters is excluded from further analysis. We compare the longest ITS estimated over a range of lag times for MSMs built

from the writhe features (red) with the largest kinetic variance (Figures S3 and S4) in addition to Euclidean distances (blue), for **(A)** α-synuclein, **(B)** ACTR, **(C)** PaaA2, **(D)** Aβ-42, **(E)** HP35 and **(F)** N_{TAIL}. After determining the optimal writhe feature set from VAMP-2 scores, the MSMs shown here are built from clustering tCCA projections using a variable number of K-means clusters and tCCA dimensions. Here, we do not explicitly enforce that MSMs be reversible in order to minimize the impact of estimator error on predicted timescales and because the sampling varies for different systems as we are utilizing publically available simulation datasets for this study. Plots excluding specific combinations of tCCA dimensions and clusters failed in estimation due to spare transitions / disconnected state spaces or numerical instability (rapid jumps) over short changes in the lag time, rendering these models invalid. Here, the selection criteria for optimal models are those with the slowest (or largest) ITS that are approximately converged with priority on model convergence (i.e., validity). We have demarcated the best performing number of tCCA dimensions hyperparameter with a asterix in the figure legends and best choices of overall models and lag times with a black star if the ITS is appropriately converged and a black ‘x’ if there are no converged ITS. We utilize 5-fold cross validated VAMP-2 scores on the resulting models to justify the choice of the number of clusters, which are shown in Figure S21.

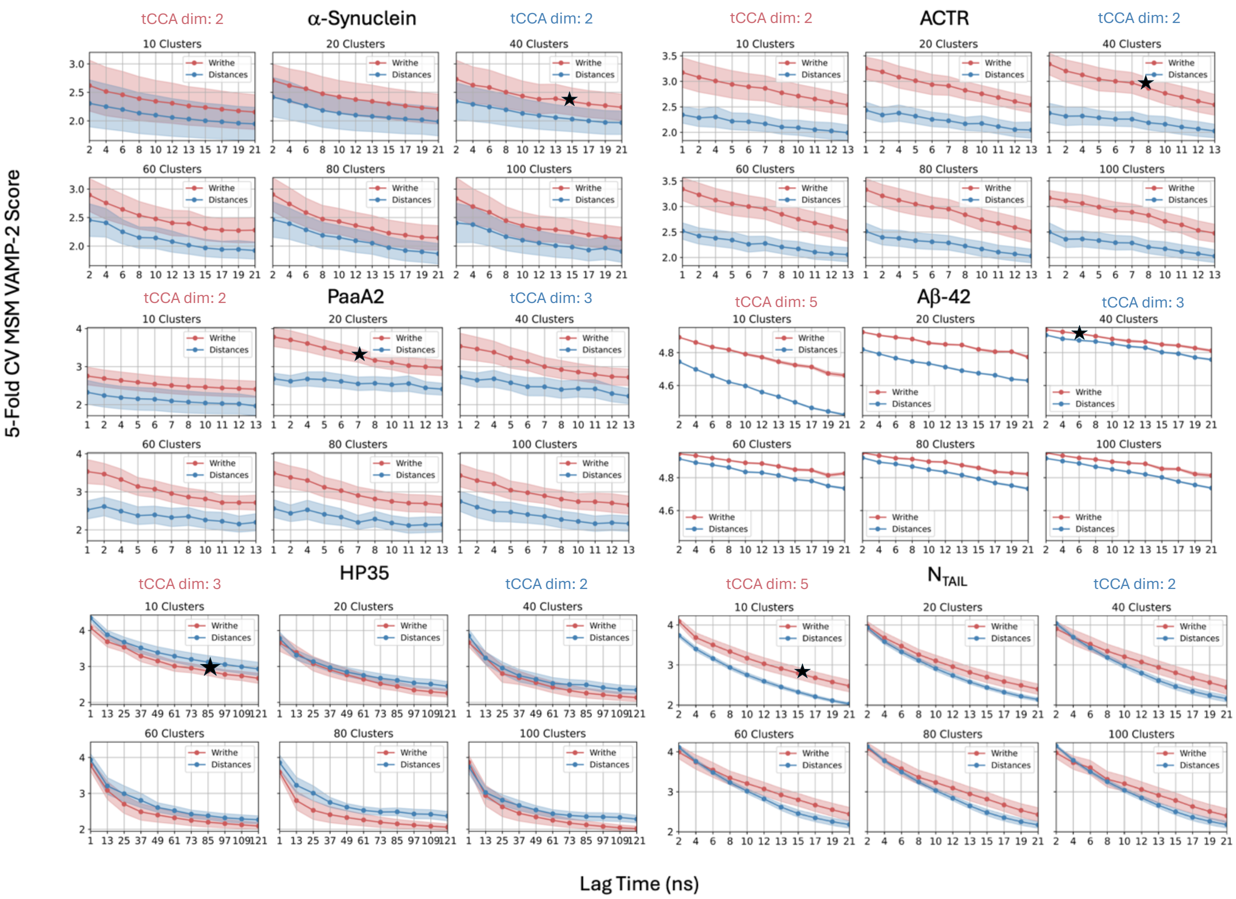

Figure S21. 5-fold cross validated MSM VAMP-2 scores estimated over a range of lag times and number of K-means clusters for MSMs built from writhe and Euclidean distance features. To further quantify the capability of writhe MSMs to capture the slowest processes observed in MD simulations, we compute cross validated (CV) MSM VAMP-2 scores over a range clusters obtained from applying K-means clustering to the best performing tCCA projection dimension as indicated in the ITS scan in Figure S20. For each system studied, we selected an optimal number of clusters by selecting the smallest value where cross validated VAMP-2 score stops increasing as a function of the number of clusters. After selecting an optimal number of tCCA dimensions and clusters, we identify the MSM with the shortest lag time that has converged to a stable ITS (Figure S20) as the best model of the dynamics of each system (indicated for writhe MSMs with a black star). A model's VAMP-2 score is only meaningful if the ITS are converged to a stable value at the lag time for which it's estimated.

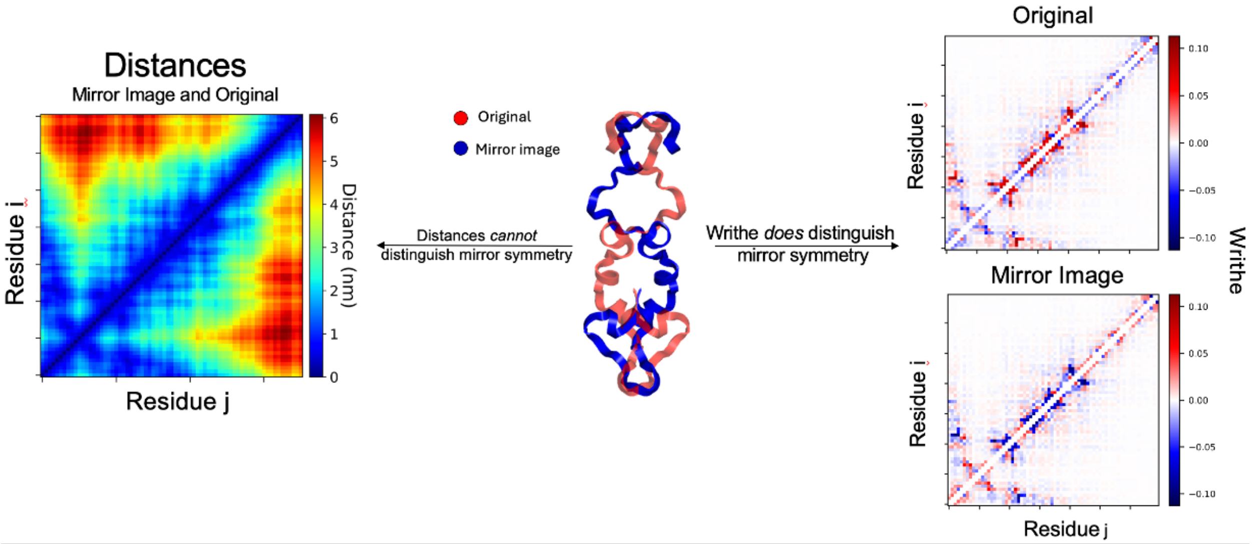

Figure S22. Properties of writhe and Euclidean distance representations.
The same Euclidean distance matrix corresponds to two structures (mirror images) that cannot be made equivalent by rotations and translations. This is because the set of all Euclidean distances for a structure is invariant to actions of the E(3) group. Thus, rotations, translations and parity transformations cannot be recovered from this representation and conformations differing by any of these transformations cannot be distinguished by Euclidean distances. This result can be shown in a rigorous mathematical sense via the relation between inner products and Euclidean distances, and Cholesky decomposition, or equivalently by well-established techniques in multidimensional scaling. In contrast, the writhe matrix (matrix of all pairwise segment crossings) differentiates mirror conformations predictably (as seen in the rightmost column) where all features are distinguished by a sign flip. More formally, this is a demonstration of the writhe's equivariance to parity.

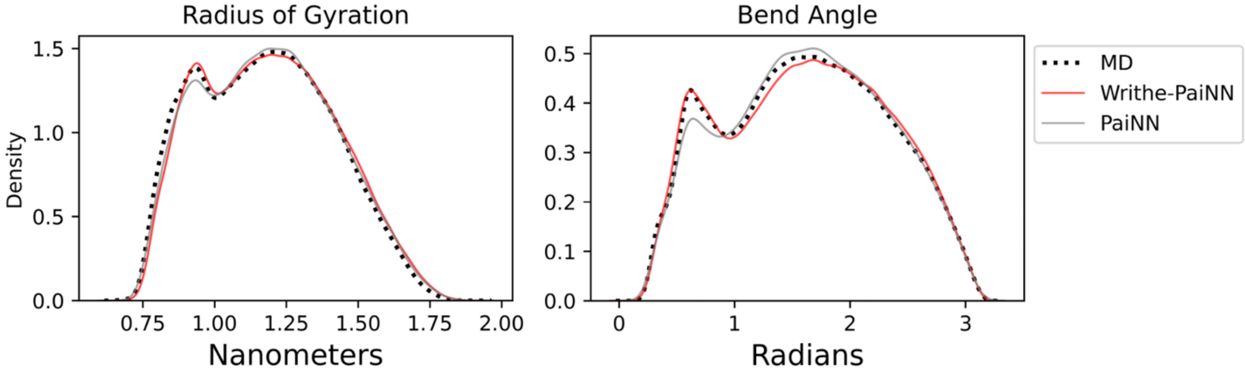

Figure S23. Distributions of parity invariant observables obtained from MD simulation and generative models. Distributions are obtained from smoothed histograms of the radius of gyration (R_g, left) and the angle formed by the first, tenth and last (twentieth) Cα atoms of the 20 residue αsynuclein fragment (right) computed directly from simulation and artificially generated atomic coordinates.

Appendix A: Numerical computation of the writhe and algorithms

The numerical computation of the writhe is characterized by the angles, $\theta_{i,j}$, formed at each vertex of the spherical quadrilateral as shown in Figure 2. The sum of these angles minus $\frac{\pi}{2}$ is equal to the surface area of the spherical quadrilateral and is referred to as the *spherical excess*. Here, we take $\vec{a}$, $\vec{b}$, and $\vec{c}$ to be normalized vectors in $\mathbb{R}^3$that represent any set of 3 view direction vectors ($\vec{d}_{i,j}$) from which we compute the contribution to the writhe from 1 of 4 vertices of the spherical quadrilateral shown in Figure 2. This is equivalent to computing the angle between $\vec{a}$ and $\vec{c}$ as seen from the direction of $\vec{b}$, minus $\frac{\pi}{2}$. The angles ($\theta_{i,j}$ in Figure 2) can be computed from cross products as:

$$\sin\left(\theta - \frac{\pi}{2}\right) = \frac{(\vec{a} \times \vec{b}) \cdot (\vec{b} \times \vec{c})}{||\vec{a} \times \vec{b}||\,||\vec{b} \times \vec{c}||} \tag{A.1}$$

where $sin^{-1}[(\vec{a} \times \vec{b}) \cdot (\vec{b} \times \vec{c})] = \theta\ - \frac{\pi}{2}$ specifically for vertices constructed following the same convention as $\vec{d}_{i,j}$ triples in Figure 2.This method of computing $\theta_{i,j}$ in Figure 2 is the most precise when the angles between $\vec{a}$, $\vec{b}$, and $\vec{c}$ are very small (< 1 degree) and should be used under single precision arithmetic constraints. Using double precision arithmetic, we can make the following simplifications to construct a faster algorithm to compute $\theta_{i,j}$. Using Lagrange's triple product identity:

$$(\vec{a} \times \vec{b}) \cdot (\vec{b} \times \vec{c}) = (\vec{a} \cdot \vec{b})(\vec{b} \cdot \vec{c}) - (\vec{a} \cdot \vec{c})(\vec{b} \cdot \vec{b}), \tag{A.2}$$

and the Gram determinant:

$$||\vec{a} \times \vec{b}|| = \sqrt{||\vec{a}||^2||\vec{b}||^2 - (\vec{a} \cdot \vec{b})^2}, \tag{A.3}$$

we can rewrite (1) in terms of dot products:

$$\sin\left(\theta - \frac{\pi}{2}\right) = \frac{(\vec{a} \times \vec{b}) \cdot (\vec{b} \times \vec{c})}{||\vec{a} \times \vec{b}||\,||\vec{b} \times \vec{c}||} = \frac{(\vec{a} \cdot \vec{b})(\vec{b} \cdot \vec{c}) - (\vec{a} \cdot \vec{c})}{\sqrt{\left(1 - (\vec{a} \cdot \vec{b})^2\right)\left(1 - (\vec{b} \cdot \vec{c})^2\right)}}, \tag{A.4}$$

where we used $|| \cdot || = 1\,, \vec{b} \cdot \vec{b} = ||\vec{b}||^2$ .We can compute the surface area (*SA*) shown in Figure 2 by evaluating (1) or (4) on the view direction vectors ($\vec{d}_{i,j}$) defining each vertex, taking the $sin^{-1}$and summing over all angles, $\theta_{i,j}$. Using equation (2) allows us to writhe the surface area (*SA*) in terms of only 6 unique dot products which we denote with Greek letters in the following:

$$\begin{aligned} &\alpha = \vec{d}_{1,3} \cdot \vec{d}_{2,3}, \quad \beta = \vec{d}_{1,3} \cdot \vec{d}_{1,4} \\ &\gamma = \vec{d}_{1,4} \cdot \vec{d}_{2,3}, \quad \sigma = \vec{d}_{1,4} \cdot \vec{d}_{2,4} \\ &\mu = \vec{d}_{1,3} \cdot \vec{d}_{2,4}, \quad \phi = \vec{d}_{2,3} \cdot \vec{d}_{2,4} \end{aligned} \implies \begin{aligned} SA =\, &sin^{-1}\frac{\alpha \cdot \beta - \gamma}{\sqrt{(1-\alpha^2)(1-\beta^2)}} + sin^{-1}\frac{\beta \cdot \sigma - \mu}{\sqrt{(1-\beta^2)(1-\sigma^2)}} \\ &+ sin^{-1}\frac{\phi \cdot \alpha - \mu}{\sqrt{(1-\phi^2)(1-\alpha^2)}} + sin^{-1}\frac{\sigma \cdot \phi - \gamma}{\sqrt{(1-\sigma^2)(1-\phi^2)}} \end{aligned} \tag{A.5}$$

Regardless of which approach is used to compute the surface area element (*SA*), the derivation of the writhe for a single crossing is completed with the same factor, $\delta_{\pm}$, which accounts for the sign of the crossing and takes on a value of 1 or -1. The sign factor can be computed from the determinant or scalar triple product of the two crossing segments, $\vec{s}_1$, $\vec{s}_2$, and the view direction vector, $\vec{d}_{1,3}$: $\delta_{\pm} = \text{sign}(\vec{d}_{1,3} \cdot (\vec{s}_2 \times \vec{s}_1)) = \text{sign}(det|\vec{d}_{1,3}\ \vec{s}_2\ \vec{s}_1|)$. From these definitions, the writhe of a crossing is:

$$\mathrm{W_r} = \frac{\delta_{\pm}}{2\pi} \cdot SA \tag{A.6}$$

The normalization constant for each individual writhe value, 2π, can be understood as $2/4\pi$ where 4π normalizes the area of the unit sphere and the factor of 2 considers that a crossing seen from one direction is also seen from the antipodal direction. Both approaches presented here are analytically correct and equivalent, however, minor discrepancies arise from floating point errors in numerical computation. Consequently, both algorithms are available in the python package accompanying this work.

Method	Dataset	Mean wall-clock time $\pm\,\sigma$ (ms)	Number of trials
Dot products	1 structure<br>140 residues<br>9,453 crossings	3.14 ± 0.066	10,000
Cross products		6.82 ± 0.093	

Table 1. **Wall-clock times to compute the writhe between all segment pairs of a single structure**. The writhe between all segment pairs was computed for a single conformation of full-length a-synuclein using a segment length of 1. Both algorithms are implemented in python using *PyTorch* and utilize broadcasting to compute the writhe of all segment pairs simultaneously. Given the number of residues in a structure, n, and the segment length, l, the number of non-trivial crossings between segments can be computed as $\frac{(n-l)^2-(n-l)-2(n-2l)}{2}$. Here, "non-trivial" refers to segment pairs that do not systematically give a writhe of zero.

Appendix B: Definition of atom-wise writhe features

To incorporate writhe into atom-wise graph neural network message-passing operations, we compute cumulative writhe features between atoms using the writhe graph Laplacian defined below. The writhe graph Laplacian operates on the graph implied by the segments (edges) connecting pairs of atoms (nodes) used to compute the writhe. The definition of the discrete computation of the writhe used in this work always implicitly defines a corresponding graph Laplacian. This allows us to systematically obtain pair-wise writhe features between atoms from pair-wise computation of the writhe between segments connecting atoms.

Here, we consider only the writhe computed at segment length 1. Thus, for n atoms, there are (n - 1) segments. The atom-segment relationships can be encoded using the incidence matrix, $B \in$

$\mathbb{R}^{n\times(n-1)}$, where rows represent atoms and columns represent segments. This incidence matrix corresponds to a graph comprised of nodes sequentially connected by undirected edges. In general, the incidence matrix, *B*, takes the form:

$$B = \begin{bmatrix} 1 & 0 & 0 & \cdots & 0 \\ 1 & 1 & 0 & \cdots & 0 \\ 0 & 1 & 1 & \cdots & 0 \\ 0 & 0 & 1 & \cdots & 0 \\ \vdots & \vdots & \vdots & \ddots & 0 \\ 0 & 0 & 0 & 0 & 1 \end{bmatrix} \tag{B.1}$$

Pairwise computation of the writhe between segments yields the symmetric writhe matrix, $W \in \mathbb{R}^{(n-1)\times(n-1)}$:

$$W = \begin{bmatrix} 0 & 0 & w_{1,3} & w_{1,4} & \cdots & w_{1,n-1} \\ 0 & 0 & 0 & w_{2,4} & \cdots & w_{2,n-1} \\ w_{1,3} & 0 & 0 & 0 & \cdots & w_{2,n-1} \\ w_{1,4} & w_{2,4} & 0 & 0 & \cdots & w_{4,n-1} \\ \vdots & \vdots & \vdots & \vdots & \ddots & \vdots \\ w_{1,n-1} & w_{2,n-1} & w_{3,n-1} & w_{4,n-1} & \cdots & 0 \end{bmatrix} \tag{B.2}$$

where each $w_{i,j}$ represents the scalar value of the writhe between segments (i, j) and

$$w_{ij} = \begin{cases} 0, & \text{if } |i - j| < 2 \\ w_{ji}, & \text{for all } i, j \end{cases} . \tag{B.3}$$

The graph Laplacian[9], $L_{wr} \in \mathbb{R}^{n\times n}$, corresponding to the incidence matrix, B, weighted by the writhe matrix, *W*, is defined as:

$$L_{wr} = BWB^T \tag{B.4}$$

and takes the general form:

$$L_{wr} = \begin{bmatrix} 0 & 0 & w_{1,3} & (w_{1,3} + w_{1,4}) & \cdots & w_{1,n-1} \\ 0 & 0 & w_{1,3} & (w_{1,3} + w_{1,4} + w_{2,4}) & \cdots & (w_{1,n-1} + w_{2,n-1}) \\ w_{1,3} & w_{1,3} & 0 & w_{2,4} & \cdots & (w_{2,n-1} + w_{3,n-1}) \\ (w_{1,3} + w_{1,4}) & (w_{1,3} + w_{1,4} + w_{2,4}) & w_{2,4} & 0 & \cdots & (w_{3,n-1} + w_{4,n-1}) \\ \vdots & \vdots & \vdots & \vdots & \ddots & \vdots \\ w_{n-1,1} & (w_{1,n-1} + w_{2,n-1}) & (w_{2,n-1} + w_{3,n-1}) & (w_{3,n-1} + w_{4,n-1}) & \cdots & 0 \end{bmatrix}$$

Each index in L_{wr} is the sum over all segment crossings in which a pair of atoms both appear and are not a part of the same segment. Programmatically, the operations described above are performed using summation operations on graphs rather than directly evaluating matrix operations.

This allows us to avoid redundant computations involving symmetric matrices and generalize the writhe graph Laplacian to the case where each w_{ij} is a vector ($\vec{w}_{i,j}$).

To demonstrate the similarity of a symmetric writhe matrix and the writhe graph Laplacian we compare the average $Wr_{l=1}$ writhe matrix computed from a 30μs MD simulation of ACTR with the average writhe graph Laplacian for the same trajectory in Figure S24.

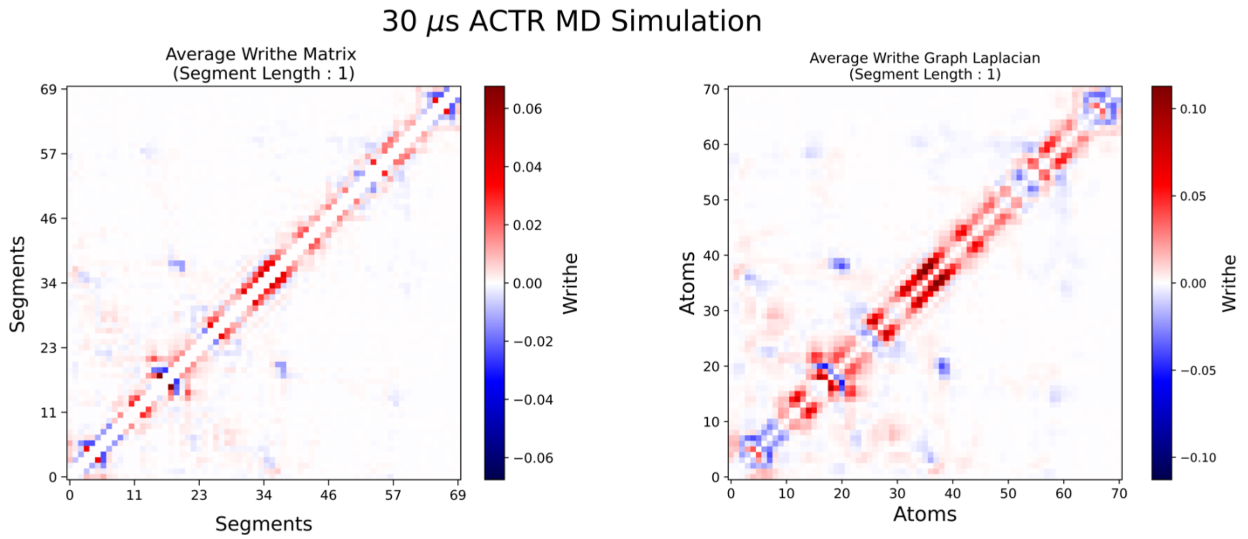

Figure S24: Comparison of the average $Wr_{l=1}$ writhe matrix and the writhe-graph Laplacian computed from a 30 μs MD simulation of ACTR.

Appendix C: PaiNN architecture implementation and DDPM training

All DDPMs were trained on structural data obtained from a 100 μs all atom molecular dynamics simulation of a 20 residue C-terminal fragment of α-synuclein[10] using the a99SB-disp protein force field[5] and a99SB-disp water model.[5] We train DDPMs on the full simulation dataset comprised of ≈550,000 structures and consider only the coordinates of Cα atoms to minimize computational expense. Both the PaiNN and Writhe-PaiNN architectures have input blocks that assign atoms and bonds trainable embedding vectors (categorical encodings) that are treated as invariant scalar features (s_i) and compute direction vectors ($\vec{r}_{i,j}$) and Euclidean distances ($\|\vec{r}_{i,j}\|$) between atoms (i, j). In the writhe model, the input block also computes pair-wise vector and scalar writhe features (Appendices A and B). The dimension of the model is a hyperparameter that determines the dimension of categorical embeddings (atom-types and bonds) and positional encodings (distances and writhe when applicable). Here, we use a model dimension of 64 in all experiments. We repeat message passing layers 8 times to construct the message passing blocks for both the PaiNN and Writhe-PaiNN. Both models are equipped with the same output block that transforms higher dimension invariant scalar (s_i) and equivariant vector (v_i) features into a prediction of the *score field*, which has the same dimension as the Euclidean coordinates of the target structure.

In Figures S25-27, we compare generated ensembles from the PaiNN and writhe-PaiNN models as a function of the training epoch using pairwise Cα distances, writhe (segment length of 1), torsion angles computed from the backbone of generated structures, RMSD from the MD simulation data and the radius of gyration. We trained models with the ADAM[11] optimizer using a learning rate of 0.0001 until the loss[12] stabilized, after which point we monitored how well generated samples from models were able to reproduce distributions from the training data. For comparison purposes, we present the results from PaiNN and Writhe-PaiNN DDPMs trained for 500 epochs and sampled 25,00 times every 25 epochs.

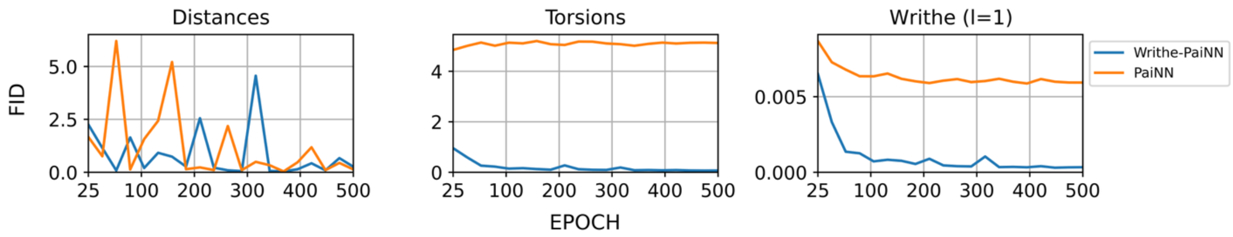

Figure S25. Comparing the Fréchet Inception Distance (FID) between generated ensembles and MD training data as a function of the training epoch. We train DDPMs using both the Writhe-PaiNN (blue) and PaiNN (orange) architectures for 500 epochs in total and generate 25,000 structure samples every 25 training epochs to monitor training progress and how well each model can reproduce the MD training data. Fréchet Inception Distances (FID) are computed for all pairwise Cα distances, backbone torsions and pairwise writhe ($Wr_{l=1}$) values, respectively.

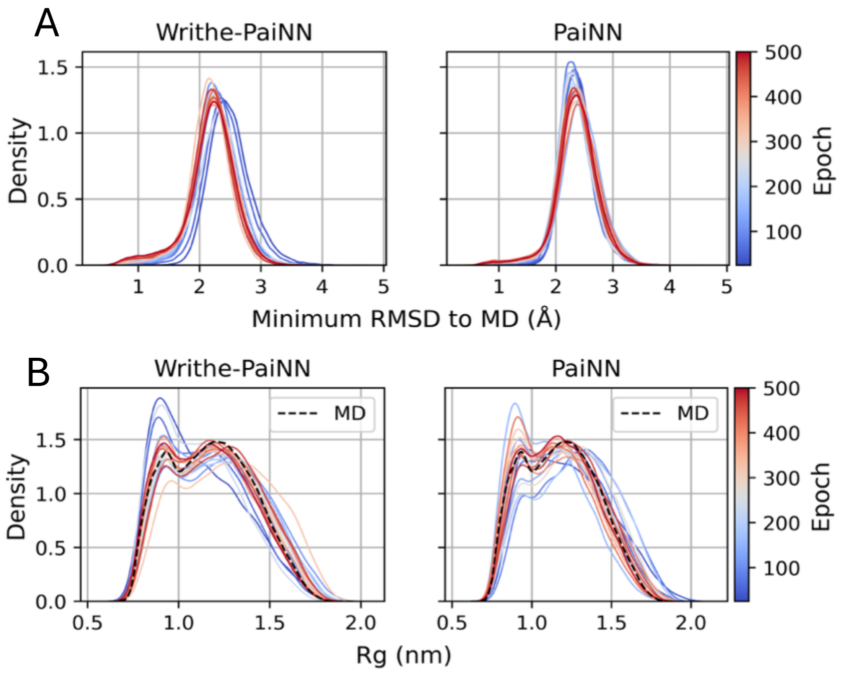

Figure S26. Comparing the similarity of generated structures and the radius of gyration to MD training data as a function of the training epoch. We train DDPMs using both the Writhe-PaiNN (left column) PaiNN (right column) architectures for 500 epochs in total and generate 25,000 structure samples every 25 training epochs to monitor training progress and how well each model can reproduce the MD training data. We quantify the similarity of generated ensembles to the MD training data using **(A)** the minimum RMSE of each generated structure to any structure in the MD ensemble and **(B)** the radius of gyration.

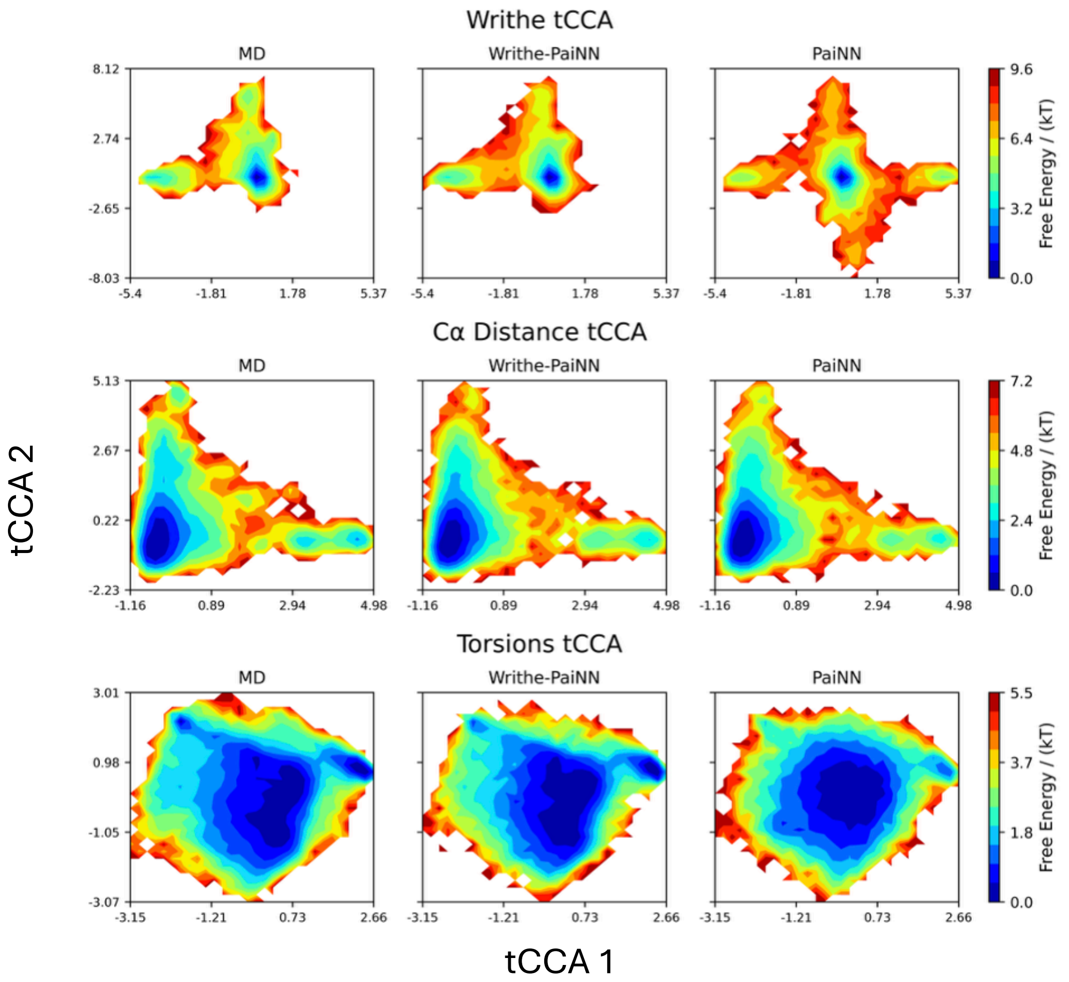

Figure S27. **Visualizing the similarity of generated structures and MD training data after 500 epochs of model training.** 50,000 structure samples were generated after 500 epochs of model training for DDPMs using the Writhe-PaiNN and PaiNN architectures, respectively. tCCA was performed on the MD training data using all pairwise Cα distances, backbone torsions and pairwise writhe ($Wr_{l=1}$) values, respectively. We compute the same sets of features for both generated ensembles and project each set onto tCCA components estimated from the MD training data. We compare the resultant 2D free energy surfaces obtained for the MD training data (left column) and generated ensembles from the Writhe-PaiNN (middle column) and PaiNN models (right column).

References

(1) Konstantin, K.; Langowski, J. Computation of writhe in modeling of supercoiled DNA. *Biopolymers* **2000**, *54* (5), 307-317.

(2) Călugăreanu, G. L'intégrale de Gauss et l'analyse des nœuds tridimensionnels. *Rev. Math. Pures Appl.* **1959**, *4*, 5–20.

(3) Røgen, P.; Bohr, H. A new family of global protein shape descriptors. *Mathematical Biosciences* **2003**, *182* (2), 167-181.

(4) Røgen, P.; Fain, B. Automatic classification of protein structure by using Gauss integrals. *Proceedings of the National Academy of Sciences* **2002**, *100* (1), 119-124.

(5) Robustelli, P.; Piana, S.; Shaw, D. E. Developing a molecular dynamics force field for both folded and disordered protein states. *Proceedings of the National Academy of Sciences* **2018**, *115* (21), E4758-E4766.

(6) Hotelling, H. Relations Between Two Sets of Variates. *Biometrika* **1936**, *28* (3/4), 321.

(7) Wu, H.; Noé, F. Variational Approach for Learning Markov Processes from Time Series Data. *Journal of Nonlinear Science* **2019**, *30* (1), 23-66.

(8) Borthakur, K.; Sisk, T. R.; Panei, F. P.; Bonomi, M.; Robustelli, P. Determining accurate conformational ensembles of intrinsically disordered proteins at atomic resolution. *bioRxiv* **2024**.

(9) Strang, G. *Linear algebra and learning from data*; Wellesley-Cambridge Press, 2019.

(10) Robustelli, P.; Ibanez-de-Opakua, A.; Campbell-Bezat, C.; Giordanetto, F.; Becker, S.; Zweckstetter, M.; Pan, A. C.; Shaw, D. E. Molecular Basis of Small-Molecule Binding to α-Synuclein. *Journal of the American Chemical Society* **2022**, *144* (6), 2501-2510.

(11) Kingma, D. P.; Ba, J. Adam: A Method for Stochastic Optimization. arXiv preprint arXiv:1412.6980: 2014.

(12) Song, Y.; Durkan, C.; Murray, I.; Ermon, S. Maximum Likelihood Training of Score-Based Diffusion Models. *arXiv preprint arXiv:2101.09258* **2021**.
